# Age, sex, residence, and region-specific differences in prevalence and patterns of multimorbidity among older Chinese: evidence from Chinese Longitudinal Healthy Longevity Survey

**DOI:** 10.1186/s12889-022-13506-0

**Published:** 2022-06-04

**Authors:** Siyue Han, Guangju Mo, Tianjing Gao, Qing Sun, Huaqing Liu, Min Zhang

**Affiliations:** 1 School of Public Health, Bengbu Medical College, Bengbu, 233030 Anhui China; 2 School of Health Management, Bengbu Medical College, Bengbu, 233030 Anhui China

**Keywords:** Ageing, China, Chronic disease, Multimorbidity, Association rule mining

## Abstract

**Background:**

Multimorbidity among older adults, which is associated with added functional decline and higher health care utilization and mortality, has become increasingly common with the dramatic acceleration of ageing in China. The purpose of this study was to reveal age, sex, residence, and region- specific prevalence and patterns of multimorbidity among older adults in China.

**Methods:**

This study is based on the 2018 Chinese Longitudinal Health Longevity Survey (CLHLS), the most recent edition of this national survey, and involved analysis of 15,275 participants aged 65 years and older. Multimorbidity was defined as an individual who has two or more chronic diseases or conditions and was divided into two types for analysis: ≥2 (MM2+) and ≥ 3 (MM3+). Fourteen chronic diseases or conditions surveyed were used to assess patterns of multimorbidity through association rule mining.

**Results:**

Among the 15,275 participants, the largest proportion (39.9%) was 90 years old and over, while the distribution of sex and residence is roughly the same. Overall, the prevalence of multimorbidity was 44.1% for MM2+ and 22.9% for MM3+. The most frequently occurring patterns were two or three combinations between hypertension, cardiovascular diseases and affective disorders. Cardiovascular diseases combined with diabetes or dyslipidemia showed the most predominant association in different age groups. Moreover, the prevalence of the hypertension +diabetes pattern decreased with age. The strongest associations were found for the clustering of hypertension + cardiovascular diseases + respiratory diseases in males, however, among females it was the cardiovascular diseases + diabetes cluster. Cardiovascular diseases + rheumatoid arthritis + visual impairment was observed in urban areas and hypertension + cardiovascular diseases + affective disorders in rural areas. The most distinctive association rule in Northern China was {cardiovascular diseases, hypertension, visual impairment} = > {diabetes}. Respiratory disease was more prevalent in combination with other systemic disorders in Western China, and affective disorders in Southern China.

**Conclusions:**

The prevalence of multimorbidity among older Chinese was substantial, and patterns of multimorbidity varied by age, sex, residence, and region. Future efforts are needed to identify possible prevention strategies and guidelines that consider differences in demographic characteristics of multimorbid patients to promote health in older adults.

**Supplementary Information:**

The online version contains supplementary material available at 10.1186/s12889-022-13506-0.

## Introduction

### Population ageing and chronic disease

Over the seventy years from 1950 to 2020, the life expectancy of the world population has increased from 47.0 years to 73.2 years [1]. In 2019, the proportion of older adults, aged 65 and over, in China reached 12.6%. The rate is increasing every year, indicating that China will become a moderately ageing society in the near future [2]. In 2016, up to 75.8% of Chinese people over the age of 60 had at least one chronic disease, amongst which the prevalence of hypertension, diabetes, and hypercholesterolemia was 58.3, 19.4, and 10.5%, respectively [3]. Common findings from global studies of chronic diseases of older adults suggest that the proportion of individuals with multiple chronic diseases at the same time increases with increasing age [4].

### Concept and prevalence of multimorbidity

The concept of multimorbidity is commonly defined as the simultaneous occurrence of two or more chronic diseases in an individual [5]. A scoping review based on a large database of studies found that most studies used two or more chronic conditions to define the presence of multimorbidity. Because there is no uniform gold standard for measuring multimorbidity, prevalence depends on the number of chronic diseases studied or the population studied. From 2012 to 2019, the prevalence of chronic multimorbidity in the current population ranged from 15.3 to 93.1% [6]. However, it has long been noted that for older patients treated in ambulatory care settings, the criteria of three chronic diseases are considered more valid cut-off points for multimorbidity than the usual criteria of two chronic diseases [7].

### Effects of multimorbidity on health

Multimorbidity has caused disabilities, functional decline, poor quality of life, and high health care utilization [8, 9]. In addition, the risk of death has significantly risen [10]. Older adults with multimorbidity attend five times more physician appointments than those without chronic conditions [10, 11]. Research showed that older adults with multiple medical conditions have the highest unmet need for hospitalization in China [12]. Although more and more people are beginning to acknowledge the seriousness of multimorbidity, the majority of health care systems focus on treating the adverse consequences of individual or major diseases, ignoring the interplay of multiple diseases, which prevents the measure from making full use of limited health resources to effectively treat and manage the multimorbidity. In contrast, patient-centered management of multimorbidity and in-depth research on the impact of multimorbidity can reduce patient treatment burden and improve patient capacity [13].

### Multimorbidity patterns and association rule mining

In recent years, an increasing number of studies have explored the multimorbidity. However, there is still lack of literature on demography-specific differences in multimorbidity patterns like age, sex, residence, and region; moreover, many studies have been limited to localized areas or descriptive analyses. General analytical methods tend to describe epidemiology and cannot determine which diseases are more strongly associated with each other. Association Rule Mining (ARM), originally introduced as a market basket analysis tool [14], is now one of the most valuable tools for performing analytical exploratory data analysis on a broad range of research, including biological and medical fields. ARM is a procedure that reveals interdependence and association between different factors, and is an important technique in data mining as it can extract valuable correlated data items from large amounts of data and reflect the degree of association. Association rules have also been widely used to mine risk factors for chronic diseases, generating rules with higher confidence and more relevant to a larger set of participants [15–17]. Based on association rules in data mining, binary and ternary patterns of multimorbidity in older adults are obtained to seek the current focus of multiple chronic diseases or conditions.

The Chinese Longitudinal Healthy Longevity Survey (CLHLS) is a national survey that covers approximately 85% of China’s population and provides representative data to investigate the determinants of longevity. It aims to fill data and knowledge gaps in scientific research and policy analysis on healthy ageing, and is considered to be high-quality because of its broad survey scope, long duration, abundant individual microdata, and robust results of reliability testing [18, 19]. Due to its large population size on healthy ageing, the CLHLS is of great value to assess patterns of multimorbidity among older Chinese, especially for the oldest old. Based on it, this study aims to explore age, sex, residence, and region-specific differences in prevalence and patterns multimorbidity patterns by the ARM method in older Chinese adults, providing a scientific basis for multimorbidity management guidelines, as well as facilitate the design and implementation of better healthcare systems for patients with multimorbidity.

## Methods

### Data source and study population

Data came from the CLHLS, a widely representative cohort survey conducted by the center for healthy ageing and family studies at Peking University and the Chinese center for disease control and prevention (http://opendata.pku.edu.cn/dataverse/CHADS). This survey was first instituted in 1998 and is conducted at roughly three-year intervals to gather information on health status, socioeconomic characteristics, lifestyle, psychological attitudes, and the accessibility of healthcare service. All information was obtained through face-to-face interviews by specially trained interviewers from the local centers for disease prevention and control. In cases where participants were unable to answer questions, a proxy respondent (usually a spouse or a close relative) was interviewed, but questions about mood were answered by the participants themselves. The CLHLS study was approved by the Research Ethics Committee of Peking University (IRB00001052–13074), and all participants or their proxy respondents provided written informed consent.

The samples were selected from 23 of 34 provinces in China. Detailed sampling procedures are available in elsewhere [18]. The most recent survey (2018) was used to explore the multimorbidity patterns in this study and involved interviews with 15,874 participants. After excluding 599 of these participants because they were under the age of 65, multiple imputation was used to deal with missing data, a valid sample of 15,275 were analyzed in this study.

### Chronic disease and multimorbidity

Eighteen chronic noncommunicable diseases or conditions in CLHLS (see Table S1 in Additional file 1) and two affective disorders were included in our study. Most chronic diseases or conditions listed in the CLHLS are determined by answering the question: “Are you currently suffering from any of the following chronic diseases?” For the purpose of our analysis, heart disease and stroke/CVD were merged into “cardiovascular disease”; bronchitis, emphysema, pneumonia, and asthma were merged into “respiratory disease”; cataract and glaucoma were merged into “vision impairment;” Parkinson’s disease, dementia, and epilepsy were merged into “nervous system disease”; arthritis and rheumatism/rheumatoid disease were merged into “rheumatoid arthritis”; and cholecystitis and cholelithiasis were merged into “biliary disease.”

Participants were identified as having an affective disorder if they had an anxiety disorder and/or depression. The Generalized Anxiety Disorder Scale (GAD-7) was used to assess anxiety symptoms and consists of seven negative-oriented questions. All responses were coded on four scales ranging from “none” (coded as 0), “a few days” (coded as 1), “more than half the time” (coded as 2), and “almost every day” (coded as 3). Total scores ranged from 0 to 21, with > 4 being diagnosed as having anxiety disorder [20]. Depression disorder was accessed by the 10-item Center for Epidemiologic Studies Depression (CES-D) scale [21], which includes eight negative-oriented questions and two positive-oriented questions. We recoded all responses in a four-scale metric, ranging from “always” (coded as 0), “often” (coded as 1), “sometimes” (coded as 2), and “seldom or never” (coded as 3). Furthermore, the positive-oriented questions, including “are you full of hope for future life?” and “do you feel as happy as you were when you are young?” were reverse coded before the summary (“seldom or never” coded as 0, “sometimes” coded as 1, “often” coded as 2, and “always” coded as 3). The depression score ranges from 0 to 30, with a higher score suggesting a greater degree of depressive symptoms. A cutoff of 10 was defined as diagnosed with depression disorder. Multimorbidity was defined as an individual who has two or more chronic diseases or conditions and was divided into two types for analysis: ≥2 (MM2+) and ≥ 3 (MM3+).

### Variables

Personal information was collected about the respondents, including age, sex, and region. Age was classified into three categories: 65–79 years, 80–89 years, and ≥ 90 years. Residences were divided into urban (city and town) and rural on the basis of their geographical location. We categorized the regions into East (Shanghai, Jiangsu, Zhejiang, Anhui, Fujian, Jiangxi, and Shandong), West (Chongqing, Sichuan, and Shaanxi), South (Guangdong, Guangxi, and Hainan), North (Beijing, Tianjin, Hebei, Shanxi, Liaoning, Jilin, and Heilongjiang) and Central (Henan, Hubei, and Hunan).

### Association rule mining (ARM)

ARM is the process of exploring associations between data items. Frequent itemset and association rules are mined according to the Apriori algorithm approach to discover combinations of variables that are associated in large databases [22, 23]. ARM consists of two steps: first, it involves listing all high-frequency items in the set; second, it generates frequent association rules based on the high-frequency items [24]. In the Apriori algorithm, Support, Confidence, and Lift are the measures of the degree of association. An association rule is an implication of the form {X} → {Y}, where {X} and {Y} are disjointed, non-empty sets of codes. Code sets of {X} and {Y} are the antecedents and the consequents, respectively. The strength of an association rule {X} → {Y} can be measured by Support (the prevalence of both X and Y co-occurring.) and Confidence (the probability that Y occurs given that X is already present.). Lift refers to the deviation of the support parameter from what would be expected if X and Y were independent; Lift (X → Y) = P(X, Y) / P(X) × P(Y). When the Lift value is > 1, it implies X and Y are positively correlated. A higher Lift value indicates a stronger association between X and Y.

Considering the CLHLS dataset, for example, we used the rule “hypertension, rheumatoid arthritis=>vision impairment.” This is expressed as follows: if a participant has both hypertension and rheumatoid arthritis (antecedent), this will lead to vision impairment (consequent). To simplify the analysis, the following minimum thresholds were used to define the degree of interest: support ≥3.0% and confidence > 30.0%, lift > 1. This means that for all association rules shown here ({X} → {Y}), the joint set {X, Y} emerges more frequently than would be expected under statistical independence, the consequent ({Y}) occurring in at least 30% of all cases that show the morbidity in the antecedent ({X}).

### Statistical analysis

In this study, missing data existed for all chronic disease variables at random, ranging from 653 in hypertension to 1097 in cancer. Direct deletion of missing data cases may cause a significant information loss, we performed multiple imputation by chained equations (MICE), using the mi package developed by Gelman in R studio [25]. Descriptive statistics were used to show sociodemographic characteristics. Analyses were stratified by age, sex, residence, and region. Categorical variables were expressed as frequencies. Frequency and percentages were reported for qualitative variables and assessed by the Chi-square test or Fisher’s exact test, as deemed appropriate. Findings at corrected *P*-values of < 0.05 were considered significant. To visualize the epidemiological trends of multimorbidity, the distribution of the study population, the prevalence of MM2+ and MM3+ was mapped using ArcMap. We also plotted percentage stacked bar charts to reflect multimorbidity. Finally, we use the arules package in R studio for ARM to identify multiple patterns of chronic diseases. Sensitive analyses were conducted for association between ADL disability and chronic conditions. Statistical analyses were conducted using SPSS, version 22 for Windows (SPSS Inc., Chicago, IL, USA), R studio, version 4.1.2 (R Foundation for Statistical Computing, Vienna, Austria), and ArcGIS, version 10.3 (ArcMap, ESRI Inc., Redlands, CA, USA).

## Results

### Chronic disease and patterns of multimorbidity

The final analytical sample included 15,275 participants, of which 5214 (34.1%) were in the 65–79 years group, 3967 (26.0%) were in the 80–89 years group, and 6094 (39.9%) were in the ≥90 years group. There was an approximately equal sex and residence distribution (44.1% males and 55.9% females, 55.6% in urban areas and 44.4% in rural areas). The distribution of the population by province in this study was shown in Fig. S1 in Additional File 1. The survey population in the 23 provinces ranged from 0.6 to 12.7%. Differences in the distribution of age, sex, and income level based on place of residence and region are presented in Table S2 in Additional File 1. In terms of income, there were significant differences by residence and region (*P*<0.001). A higher percentage of older adults in urban and northern China have total annual household income>RMB 30,000 yuan.

**Fig. 1:**
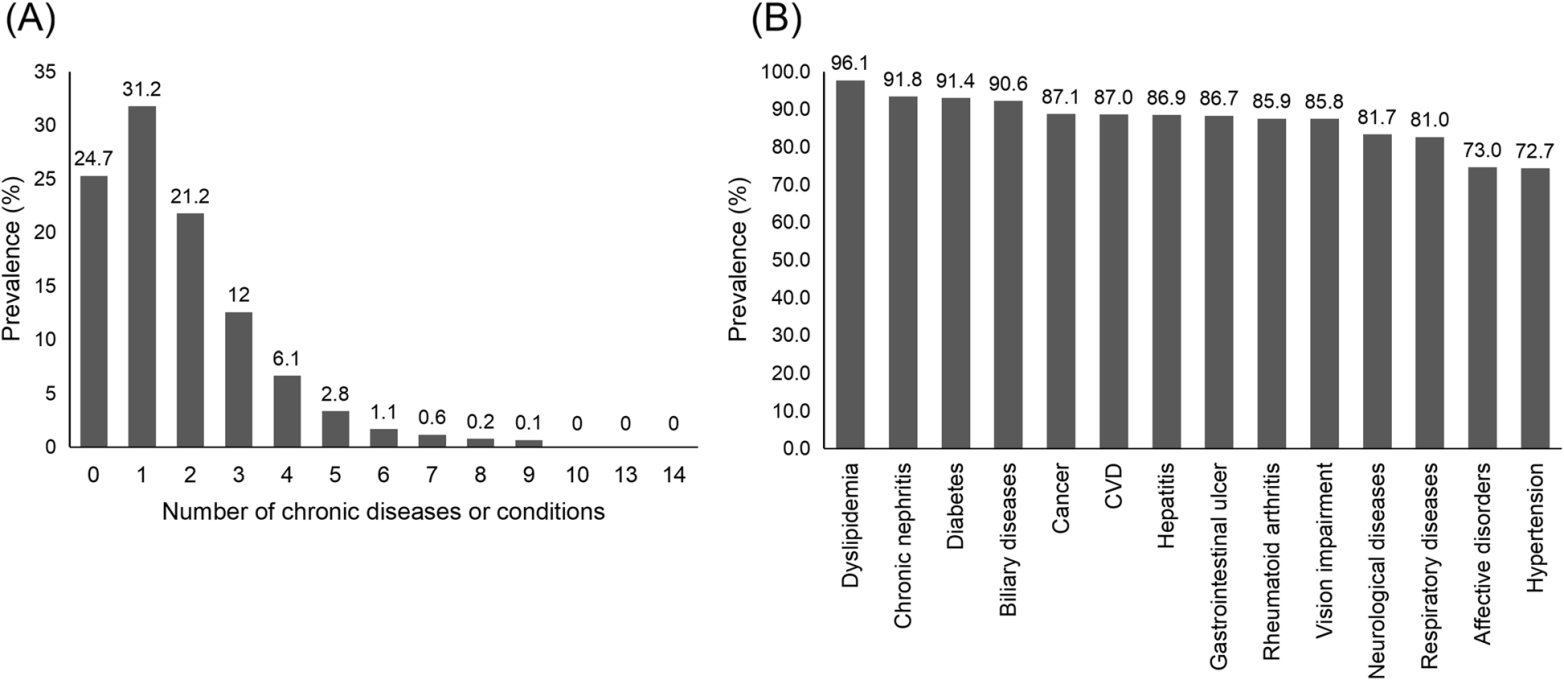
The prevalence of the number of multimorbidities (**A**), and the multimorbidity of the 14 chronic diseases or conditions in the CLHLS (**B**)

Summary multimorbidity data were shown in Fig. 1(A) and (B). The prevalence of MM2+ and MM3+ were 44.1 and 22.9%, respectively; while 31.2% had one and 24.7% had no morbidities for older Chinese adults. The total number of medical conditions present in any one individual ranged from 0 to 14. Figure 1B indicated the prevalence of multimorbidity for the 14 chronic diseases or conditions among the total population. Multimorbidity above 90% were obtained for dyslipidemia (96.1%), chronic nephritis (91.4%), diabetes (91.4%), and biliary disease (90.6%). Hypertension had the lowest multimorbidity rate (72.7%).

**Fig. 2:**
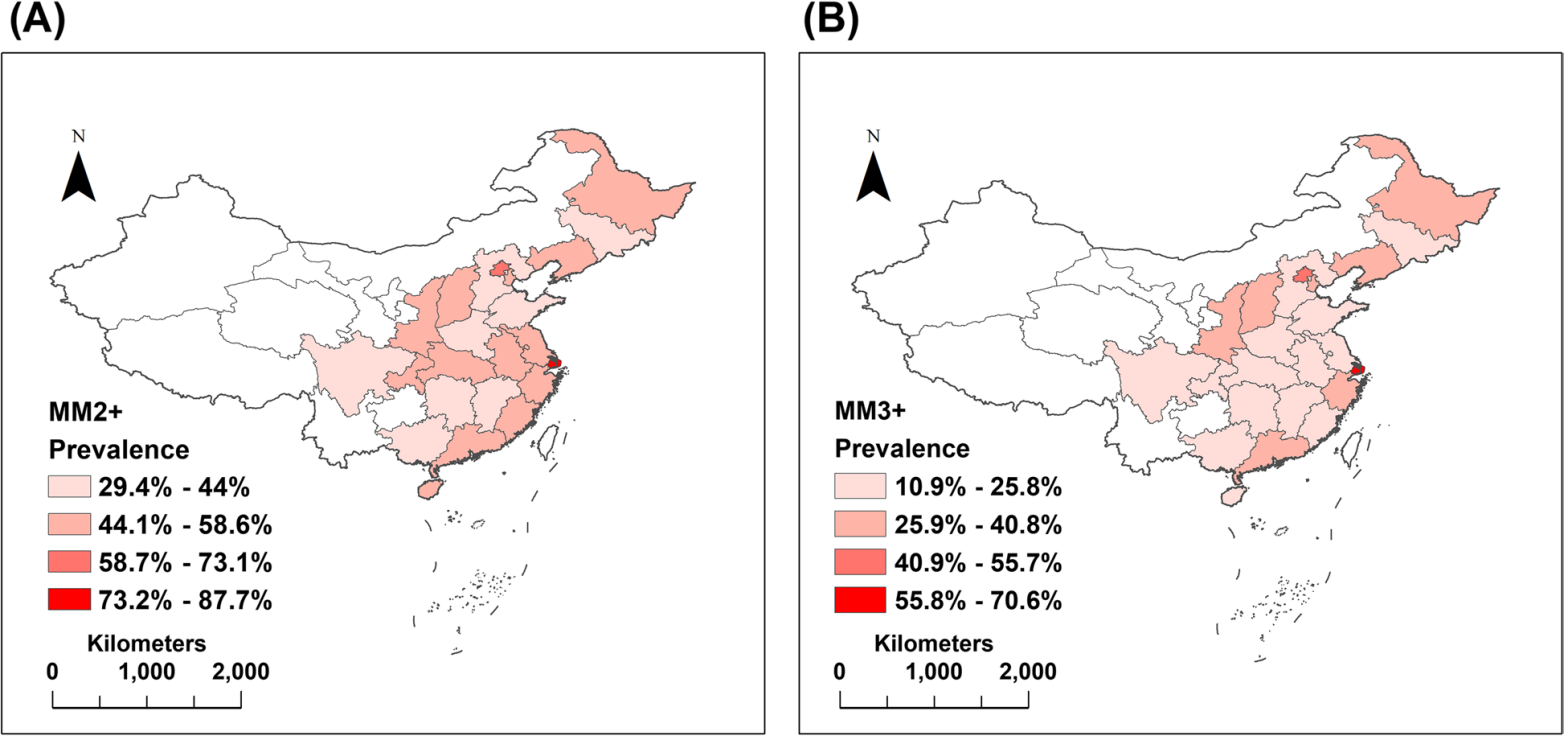
Geographical distribution of the prevalence of multimorbidity in the CLHLS. (**A**) MM2+ (**B**) MM3+

Figure 2(A) showed the distribution of MM2+. The prevalence of MM2+ in all provinces ranged from 29.4 to 87.7%. Shanghai, Beijing, Heilongjiang, Liaoning, and Guangdong had the top five MM2+ prevalence rates. The prevalence of MM2+ was generally higher in Northern China, followed by the eastern regions, and lowest in the southern regions. The analysis of the prevalence of MM3+ was shown in Fig. 2(B). Shanghai, Beijing, Heilongjiang, Tianjin, and Shanxi had the highest prevalence. The spatial distribution pattern of the prevalence of diabetes was similar to that of MM2 + .

Table 1 shows the prevalence of 14 chronic diseases or conditions of participants, as well as the specific distribution by age, sex, residence, and region. Overall, 75.3% of all participants had one or more morbidities. The prevalence of hypertension had the highest prevalence (42.8%), followed by affective disorders (28.2%) and cardiovascular disease (24.8%). The crude prevalence of diabetes, dyslipidemia, gastrointestinal ulcer, biliary disease, cancer, and hepatitis was highest in the “youngest” older group (65–79 years).

**Table 1:** Age-, sex-, residence-, and region -specific prevalence of 14 chronic diseases or conditions of participants

Chronic disease or condition	Total	Age			***P***-value	Sex		***P***-value	Residence			Region					***P***-value
		65–79 years (***n*** = 5214)	80–89 years (***n*** = 3967)	≥ 90 years (***n*** = 6094)		Male (***n*** = 6740)	Female (***n*** = 8535)		Urban (***n*** = 8491)	Rural (***n*** = 6784)	***P***-value	East (***n*** = 6224)	West (***n*** = 1993)	South (***n*** = 2994)	North (***n*** = 1599)	Central (***n*** = 2465)	
Hypertension	6538(42.8)	2468(47.3)	1935(48.8)	2135(35.0)	< 0.001	2762(41.0)	3776(44.2)	< 0.001	3905(46.0)	2633(38.8)	< 0.001	2943(47.3)	773(38.8)	1191(40.0)	763(47.7)	868(35.2)	< 0.001
Affective disorders	4302(28.2)	1370(26.3)	1219(30.7)	1713(28.1)	< 0.001	1718(25.5)	2584(30.3)	< 0.001	2369(27.9)	1933(28.5)	0.428	1796(28.9)	550(27.6)	918(30.7)	339(21.2)	699(28.4)	< 0.001
Cardiovascular diseases	3781(24.8)	1358(26.0)	1153(29.1)	1270(20.8)	< 0.001	1649(24.5)	2132(25.0)	0.465	2437(28.7)	1344(19.8)	< 0.001	1721(27.7)	404(20.3)	468(15.6)	597(37.3)	591(24.0)	< 0.001
Vision impairment	2183(14.3)	590(11.3)	626(15.8)	967(15.9)	< 0.001	821(12.2)	1362(16.0)	< 0.001	1479(17.4)	704(10.4)	< 0.001	885(14.2)	257(12.9)	365(12.2)	391(24.5)	285(11.6)	< 0.001
Rheumatoid arthritis	2065(13.5)	796(15.3)	616(15.5)	653(10.7)	< 0.001	731(10.8)	1334(15.6)	< 0.001	1236(14.6)	829(12.2)	< 0.001	745(12.0)	322(16.2)	464(15.5)	257(16.1)	277(11.2)	< 0.001
Respiratory diseases	1550(10.1)	455(8.7)	480(12.1)	615(10.0)	< 0.001	857(12.7)	693(8.1)	< 0.001	960(11.3)	590(8.7)	< 0.001	649(10.4)	283(14.2)	218(7.3)	164(10.3)	236(9.6)	< 0.001
Diabetes	1447(9.5)	695(13.3)	439(11.1)	313(5.1)	< 0.001	609(9.0)	838(9.8)	0.101	1037(12.2)	410(6.0)	< 0.001	654(10.5)	180(9.0)	192(6.4)	268(16.8)	153(6.2)	< 0.001
Dyslipidemia	742(4.9)	390(7.5)	210(5.3)	142(2.3)	< 0.001	311(4.6)	431(5.0)	0.214	557(6.6)	185(2.7)	< 0.001	369(5.9)	85(4.3)	59(2.0)	160(10.0)	69(2.8)	< 0.001
Gastrointestinal ulcer	654(4.3)	261(5.0)	196(4.9)	197(3.2)	< 0.001	281(4.2)	373(4.3)	0.542	391(4.6)	263(3.9)	0.030	330(5.3)	66(3.3)	97(3.2)	59(3.7)	102(4.1)	< 0.001
Biliary diseases	593(3.9)	244(4.7)	168(4.2)	181(3.0)	< 0.001	214(3.2)	379(4.4)	< 0.001	396(4.7)	197(2.9)	< 0.001	277(4.5)	111(5.6)	42(1.4)	75(4.7)	88(3.6)	< 0.001
Neurological diseases	471(3.1)	60(1.1)	106(2.7)	305(5.0)	< 0.001	166(2.5)	305(3.6)	< 0.001	278(3.3)	193(2.8)	0.140	195(3.1)	75(3.8)	63(2.1)	61(3.8)	77(3.1)	0.004
Cancer	201(1.3)	84(1.6)	59(1.5)	58(1.0)	0.005	105(1.6)	96(1.1)	0.020	140(1.6)	61(0.9)	< 0.001	108(1.7)	19(1.0)	15(0.5)	41(2.6)	18(0.7)	< 0.001
Chronic nephritis	147(1.0)	60(1.1)	49(1.2)	38(0.6)	0.002	76(1.1)	71(1.4)	0.063	101(1.2)	46(0.8)	0.002	49(0.8)	18(0.9)	31(1.0)	34(2.1)	15(0.6)	< 0.001
Hepatitis	61(0.4)	38(0.7)	11(0.2)	12(0.1)	< 0.001	34(0.5)	27(0.8)	0.067	38(0.4)	23(0.3)	0.354	27(0.4)	9(0.5)	11(0.4)	7(0.4)	7(0.3)	0.864
≥ 1 disease or condition	11,500(75.3)	4025(77.2)	3196(80.6)	4279(70.2)	< 0.001	4990(74.0)	6510(76.3)	0.002	6608(77.8)	4892(72.1)	< 0.001	4797(77.1)	1489(74.7)	2134(71.3)	1287(80.5)	1793(72.7)	< 0.001
Multimorbidty (2+)	6737(44.1)	2398(46.0)	1998(50.4)	2341(38.4)	< 0.001	2805(41.6)	3932(46.1)	< 0.001	4116(48.5)	2661(39.2)	< 0.001	2915(46.8)	859(43.1)	1138(38.0)	865(54.1)	960(38.9)	< 0.001
Multimorbidty (3+)	3506(23.0)	1322(25.4)	1087(27.4)	1097(18.0)	< 0.001	1407(20.9)	2099(24.6)	< 0.001	2303(27.1)	1203(17.7)	< 0.001	1565(25.1)	444(22.3)	535(17.9)	521(32.6)	441(17.9)	< 0.001

Significant at *P*- value< 0.05 by Chi-square/ Fisher Exact test

Stratification results by age group indicates that the prevalence of the 14 chronic diseases was lowest in older adults aged 90 and over, except for vision impairment and neurological diseases. Differences were observed in the prevalence of chronic diseases or conditions among age groups (*P*<0.05). Compared with females, males had a significantly higher prevalence of respiratory disease and cancer (*P*<0.001). There were no sex differences in cardiovascular disease, diabetes, dyslipidemia, gastrointestinal ulcer, chronic nephritis, and hepatitis (*P*>0.05). With the exception of affective disorders, the prevalence of the remaining 13 chronic diseases was higher in urban areas than in rural areas. The prevalence of diabetes and dyslipidemia in urban areas was actually twice as high as in rural areas (*P*<0.001). No residence differences were observed for cardiovascular disease and hepatitis (*P*>0.05). The North and South were the regions with the highest (54.1%) and lowest (38.0%) rates of multimorbidity, respectively.

The prevalence of gastrointestinal ulcer was significantly higher in Eastern China (*P*<0.001). In contrast, the prevalence of rheumatoid arthritis, respiratory diseases and biliary diseases was significantly higher in the West region (*P*<0.001). Compared with other regions, the South had a significantly higher prevalence of affective disorder (*P*<0.001). The remaining eight diseases had the highest prevalence in the North region (*P*<0.001). No region-specific differences were observed for hepatitis (*P*>0.05). Multimorbidity, both MM2+ and MM3+, was more common among those aged 80–89 years, females, and those living in urban and northern areas (*P*<0.001).

Table 2 shows the 10 most prevalent binary and ternary combinations in the total cohort. The top 3 binary multimorbidity patterns were hypertension + cardiovascular disease (16.5%), hypertension + affective disorders (14.1%), and cardiovascular disease + affective disorders (9.6%). The highest prevalence was found in ternary patterns for the combination hypertension + cardiovascular disease + affective disorders (6.4% of the total participants). A closer exploration of these 10 triadic combinations revealed that hypertension was present in each pattern.

**Table 2:** Top ten in combination clusters with multimorbidity

Rank	Dyads of morbidity		Triads of morbidity	
	Combination	***n*** (%)	Combination	***n*** (%)
1	HTN + CVD	2516(16.5)	HTN + CVD + AD	975(6.4)
2	HTN + AD	2149(14.1)	HTN + CVD + VI	682(4.5)
3	CVD + AD	1473(9.6)	HTN + CVD + DM	663(4.3)
4	HTN + VI	1341(8.8)	HTN + CVD + RA	646(4.2)
5	HTN + RA	1254(8.2)	HTN + AD + RA	526(3.4)
6	HTN + DM	1233(8.0)	HTN + AD + VI	482(3.2)
7	CVD + VI	962(6.3)	HTN + VI + RA	471(3.1)
8	AD + RA	924(6.0)	HTN + AD + DM	470(3.1)
9	CVD + RA	896(5.9)	HTN + CVD + RD	464(3.0)
10	HTN + RD	824(5.4)	HTN + CVD + DL	409(2.7)

*Abbreviations*: *HTN* hypertension, *CVD* cardiovascular diseases, *AD* affective disorders, *VI* vision impairment, *RA* rheumatoid arthritis, *DM* diabetes, *RD* respiratory diseases, *DL* dyslipidemia

The 10 most common patterns of binary and ternary combinations by age, sex, residence, and region were listed (see Additional File 2 for complete data for different subgroups). Both the prevalence (10.3%. vs. 9.7%. vs. 4.9%.) and ranking (3rd. vs. 6th. vs. 10th.) of the combination of hypertension + diabetes decreased with age. The difference between males and females was mainly the combination of the top ten male binary portfolios included combinations of respiratory disease, while the female binary portfolios did not. The prevalence of hypertension + affective disorders was highest among rural older adults and the combination associated with affective disorders was significantly higher than in urban areas, accounting for 4/10. Overall, the prevalence of binary combinations was all higher in Norther China than in other regional participants. We also found there were no combinations of diabetes in the top 10 binary combinations in the South. The dichotomous combination of respiratory diseases occurred only in the East and West. Hypertension and affective disorder ranked first in prevalence in the South and Central. In the North, however, this combination ranked fifth.

The results of the ternary multimorbidity portfolio showed that older male adults aged 65–79 years, living in urban areas in the East and North reported the most chronic diseases, with a total of eight. The combination of visual impairment or rheumatoid arthritis (degenerative diseases) with other chronic diseases or conditions increased with age in a triadic multimorbidity pattern, accounting for 7/10 in the group of participants aged ≥90 years. The prevalence of hypertension-cardiopulmonary (cardiovascular disease and respiratory disease) pattern was higher in males (3.3%), ranking fourth, while females demonstrated a higher prevalence of degenerative diseases. No significant differences were observed between the urban and rural triads. Respiratory disease was more frequent in the West than East, and absent in the South, North, and Central. Affective disorder is more prominent in the South and Central regions.

### Association rules of chronic disease

After conducting ARM, we obtained the association rule results by age, sex, residence, and region (see Additional File 3 for complete data for different subgroups). Table 3 presents the 10 rules with the strongest associations in the total population, with three binary chronic disease combinations and seven ternary chronic disease combinations. The rule {hypertension, rheumatoid arthritis} = > {visual impairment} implies that visual impairment occurred 2.39 times more frequently in older people with both hypertension and rheumatoid arthritis than we would expect under statistical independence, with the result that {visual impairment} occurred in 37.6% of cases showing a preceding incidence of {hypertension, rheumatoid arthritis}.

**Table 3:** The 10 rules with the strongest associations in the total population

Rules	Support	Confidence	Lift
{Hypertension, Rheumatoid arthritis} = > {Vision impairment}	3.1%	37.6%	2.39
{Hypertension, Vision impairment} = > {Rheumatoid arthritis}	3.1%	35.1%	2.35
{Dyslipidemia} = > {Cardiovascular diseases}	3.3%	59.9%	2.32
{Hypertension, Respiratory diseases} = > {Cardiovascular diseases}	3.0%	56.3%	2.18
{Diabetes, Hypertension} = > {Cardiovascular diseases}	4.3%	54.2%	2.10
{Rheumatoid arthritis} = > {Vision impairment}	4.8%	32.4%	2.06
{Vision impairment} = > {Rheumatoid arthritis}	4.8%	30.8%	2.06
{Hypertension, Rheumatoid arthritis} = > {Cardiovascular diseases}	4.2%	51.5%	2.00
{Cardiovascular diseases, Diabetes} = > {Hypertension}	4.3%	85.9%	1.98
{Hypertension, Vision impairment} = > {Cardiovascular diseases}	4.5%	50.9%	1.97

The results showed that there were strong association rules ranging from 16 to 99 for different subgroups, with the most in the North and the least in rural areas. The strongest association for each age group was the cardiovascular-metabolic disease (diabetes or dyslipidemia) pattern. The hypertension–cardiopulmonary pattern was most strongly associated in men, while in women it was cardiovascular–metabolic disease (dyslipidemia) and cardiovascular disease–degenerative diseases (vision impairment and rheumatoid arthritis). For different residences, the strongest urban association is for the cardiovascular–degenerative diseases (vision impairment and rheumatoid arthritis) pattern and the strongest rural association is for hypertension–cardiovascular–affective disorder.

Degenerative diseases (vision impairment and rheumatoid arthritis) were the main pattern indicating strong association in the East. Strong association rules were more predominant in the West for respiratory disease (5/32) and in the South for affective disorder (12/27). There were 99 strong association rules in the North, 70 including three or more diseases. One of the more distinctive patterns is that of hypertension–metabolic–degenerative disease, with the rule of {cardiovascular disease, hypertension, visual impairment} = > {diabetes}. Dyslipidemia was not included in the strong association rule for the South and Central areas.

## Discussion

This study examined the prevalence and trends of multimorbidity in older adults by age, sex, residence, and region based on a large national dataset in China. It is evident that chronic and multi-morbidity is a prevailing problem among China’s older adults. The study consisted of 14 relatively comprehensive chronic diseases or conditions including physical and mental illnesses (20 before systematic classification), which are a reliable basis for exploring chronic diseases and their multimorbidity patterns.

The findings indicated that 44.1% of the population suffered from multimorbidity (MM2+). Multimorbidity involving three or more conditions (MM3+) affected 22.9% of participants. The results are similar to another Chinese study that investigated 14 self-report-based chronic diseases, which found the prevalence of multimorbidity to be 42.4% in people aged 50 and over [26]. A Canada analysis included 14 common conditions, which were based on physician’ s diagnosis, defining multimorbidity as the presence of at least two conditions, with an estimated prevalence of 33.0% in adults aged 50 years and older [27]. Further, in a German study investigating 17 self-reported chronic conditions, the prevalence of defining multimorbidity as three or more conditions was 39.6% (95% confidence interval (CI) 38.7–40.6%) [28]. The reason for the discrepancy between our study and the Irish and German studies may be attributed to the higher sample size of long-lived older adults in our database (39.9%) and their lowest prevalence (38.4%). Furthermore, it is difficult to compare the prevalence rates generated due to the various types of chronic diseases and definitions of multimorbidity selected for the different studies and the varying demographic characteristics of the samples.

Our study further assessed multimorbidity patterns by age group, especially for those aged 90 years and over, a pattern that has not been mentioned in other studies. Some studies on multimorbidity have indicated that multimorbidity rates increase with age [29, 30]. Interestingly, the prevalence of multimorbidity increased with age in our study for those aged 65–89 years but ceased to increase when it reached 90 years and above. Conversely, the lowest prevalence of multimorbidity (36.4%) was observed for those aged ≥90 years. A cross-sectional study in Taiwan reported similar findings to ours in terms of trends in multimorbidity in different age groups (53.1% vs. 64.3% vs. 45.7%) [31]. In addition, a multi-country population-based study demonstrated that South Africa showed a unique pattern of a progressive decline in the prevalence of multimorbidity among adults aged 65 and older. Poland, as well as Mexico, is also beginning to see a decline in the prevalence of multimorbidity in older adults aged 70 and above [32]. This gap is likely associated with the fact that the largest proportion of the population in our study was over 90 years of age, and that the relatively lower prevalence of chronic diseases or conditions was observed among these older people, which contributed to the discrepancy. It is also possible that older people with multiple chronic conditions have died or are reluctant to participate in this survey. In addition, many participants aged 90 or over do not even have access to a definitive diagnosis of chronic diseases due to financial and physical constraints.

By measuring the psychological status of older people, we have found that affective disorders in older adults are more prevalent in rural areas of the South and Central regions. Previous study has shown that suffering from multiple mental-physical disorders is often attributed with decreased levels of functioning, and that poor quality of life and worsens as the number of conditions increases [33]. Future studies should investigate the role of physical disorders in association with clusters of multimorbid chronic diseases. In our study, adults aged 65–79 years had the highest prevalence of the hypertension–diabetes, which was trending younger. A recent study has shown that the overall rise in risk factors such as irrational diet, smoking and alcohol consumption among Chinese adults has led to a serious disease burden of diabetes and an urgent need for greater intervention [34]. The slight sex differences found in this study regarding the multimorbidity of chronic diseases are consistent with Van den et al.’s findings [28].

All combinations of cardiovascular diseases-metabolic diseases (diabetes and dyslipidemia)–degenerative diseases (rheumatoid arthritis and visual impairment) are more common in females, while cardiopulmonary diseases are more frequent in males, suggesting that a sex-specific disease spectrum for chronic disease management should be constructed for purposes of chronic disease control and intervention. We also found that the North had the highest rate of multimorbidity, the greatest number of association rules and a more abundant combination of binary and ternary diseases. This may be due to the high-sodium diet, geographic and environmental factors affecting older people in the North. The higher number of high-income households in the northern region of this study may also have a more disruptive lifestyle [35, 36]. Severe air pollution and malnutrition in the population have contributed to a pattern of cardiopulmonary disease that is more common in the West [37]. The reasons for the predominance of combinations of comorbid affective disorders in the South and Central regions have not been documented and their causes need to be explored in depth.

We observed that although results showed that the prevalence of dyslipidemia is the eighth highest among the 14 chronic diseases, it has the highest multimorbidity rate, which means that older people with dyslipidemia are at greater risk of having other complications. There are common behavioral risk factors among the diseases, e.g., poor dietary habits are risk factors for diabetes and can also lead to cardiovascular disease, hitting upon the need to change poor lifestyle before the age of 65 [12].

The main strength of our study is its adequate and representative sample size for investigating multimorbidity patterns among China’s older population living in areas with observed longevity. The study also entails analysis of multimorbidity patterns in five regions based on the participants’ current province of residence, an analysis that has rarely been conducted in previous studies.

There are several limitations to be noted in our study. Firstly, the prevalence of multimorbidity often varies widely among different studies due to the number of diseases included. A list of at least 12 chronic diseases was suggested because not much variation was observed with fewer [38]. Although we used 14 chronic diseases or conditions, which is a sufficient number to evaluate the prevalence and patterns of multimorbidity in this study, variations might still exist. Secondly, chronic diseases or conditions in CLHLS were self-reported rather than medical records; moreover, affective disorders were measured by the GAD-7 and CES-D 10 scales rather than authoritative clinical diagnoses, which is also related to the weak capacity of mental health services in China. Although the GAD-7 and CES-D 10 scales have been validated to use as tools for screening anxiety and depression in primary care [39, 40], they may overestimate the prevalence of affective disorders. Thirdly, although the CLHLS covered 23 of 34 provinces in China, which was a good representation of the older adults in China, the sample regional representativeness is relatively low for provinces in Western China. Moreover, the data used in this study included a large number of oldest old and the longevous, which is likely to cause discrepancies in multimorbidity pattern. Therefore, the results of this study might not apply to general older adults. Lastly, our study used ARM patterns of multimorbidity without regression analysis of influences on multimorbidity, and this requires further study of the influences associated with multimorbidity.

## Conclusion

The multimorbidity of chronic diseases among older adults in China will become increasingly severe and complex as the population ages, resulting in an increased difficulty in multimorbidity management. The fact that an individual can suffer from more than two chronic diseases underlies the need to take integrated measures to simultaneously treat and manage increasing multiple and coexisting chronic diseases. Existing practice guidelines tend to manage single chronic diseases in isolation, failing to meet the demand for therapy and management of chronic diseases under different multimorbidity patterns. This study on multimorbidity patterns of chronic disease and screening for high prevalence patterns helps provide an entry point for research into multimorbidity etiology and serves as a reference for the subsequent development of treatment guidelines for high prevalence multimorbidity combinations, along with disease prevention and health management.

## Supplementary Information

**Additional file 1: Table S1**. Chronic diseases orconditions list for the 2018 survey. **Table S2.** Characteristic of the study participants according to residence and region. **Figure S1.** Geographical distribution of the CLHLS study population.

**Additional file 2: Table S1.** Top ten frequent unique combination clusterswith multimorbidity, stratified by age. **Table S2.** Top ten frequentunique combination clusters with multimorbidity, stratified by sex. **Table S3.** Top ten frequent unique combination clusters with multimorbidity, stratified by residence. **Table S4.** Top ten frequent unique combination clusters with multimorbidity, stratified by region.

**Additional file 3: Table S1.** Analysis results of the association rules in the total population. **Table S2.** Analysis results of the association rules for 65–79-year-olds. **Table S3.** Analysis results of the association rules for 80–89-year-olds. **Table S4.** Analysis results of the association rules for ≥90-year-olds. **Table S5.** Analysis results of the association rules for males. **Table S6.** Analysis results of the association rules for females. **Table S7.** Analysis results of the association rules in urban areas. **Table S8.** Analysis results of the association rules in rural areas. **Table S9.** Analysis results of the association rules in the East. **Table S10.** Analysis results of the association rules in the West. **Table S11.** Analysis results of the association rules in the South. **Table S12.** Analysis results of the association rules in the North. **Table S13.** Analysis results of the association rules in the Central.

## Data Availability

Data are from the 2018 Chinese Longitudinal Healthy Longevity Survey, which is a public, open access repository (https://opendata.pku.edu.cn).

## Abbreviations

CLHLS: Chinese Longitudinal Longevity Survey (CLHLS)
ARM: Association Rule Mining
HTN: Hypertension
CVD: Cardiovascular disease
AD: Affective disorder
VI: Vision impairment
RA: Respiratory disease
DM: Diabetes
RD: Respiratory disease
DL: Dyslipidemia
