# Supplementary material for: Age, sex, residence, and region-specific differences in prevalence and patterns of multimorbidity among older Chinese: evidence from Chinese Longitudinal Healthy Longevity Survey
Source: BMC Public Health. 2022 Jun 4;22:1116. doi: 10.1186/s12889-022-13506-0 (PMC9166487; doi:10.1186/s12889-022-13506-0)
Supplement: Supplementary file 1 — Additional file 1: Table S1. Chronic diseases orconditions list for the 2018 survey. Table S2. Characteristic of the study participants according to residence and region. Figure S1. Geographical distribution of the CLHLS study population. [file 12889_2022_13506_MOESM1_ESM.docx]

**Supplementary Appendix**

**Table S1.** Chronic diseases or conditions list for the 2018 survey.

**Figure S1.** Geographical distribution of the CLHLS study population.

**Table S2.** Characteristic of the study participants according to residence and region.

**Table S1** Chronic diseases or conditions list for the 2018 survey.

| **Questions** | **Response** |
| --- | --- |
| Are you suffering from hypertension? | 1.Yes; 2.No |
| Are you suffering from from diabetes? | 1.Yes; 2.No |
| Are you suffering from heart disease? | 1.Yes; 2.No |
| Are you suffering from stroke, cerebrovascular disease? | 1.Yes; 2.No |
| Are you suffering from bronchitis, emphysema, pneumonia, asthma? | 1.Yes; 2.No |
| Are you suffering from pulmonary tuberculosis? | 1.Yes; 2.No |
| Are you suffering from cataracts? | 1.Yes; 2.No |
| Are you suffering from glaucoma? | 1.Yes; 2.No |
| Are you suffering from cancer? | 1.Yes; 2.No |
| Are you suffering from gastric or duodenal ulcer? | 1.Yes; 2.No |
| Are you suffering from Parkinson’s disease? | 1.Yes; 2.No |
| Are you suffering from arthritis? | 1.Yes; 2.No |
| Are you suffering from dementia? | 1.Yes; 2.No |
| Are you suffering from epilepsy? | 1.Yes; 2.No |
| Are you suffering from cholecystitis, cholelith disease? | 1.Yes; 2.No |
| Are you suffering from dyslipidemia? | 1.Yes; 2.No |
| Are you suffering from rheumatism or rheumatoid disease? | 1.Yes; 2.No |
| Are you suffering from chronic nephritis? | 1.Yes; 2.No |
| Are you suffering from hepatitis? | 1.Yes; 2.No |

**Figure S1.** Geographical distribution of the CLHLS study population.

**Table S2.** Characteristic of the study participants according to residence and region.

|  | **Residence** | | ***P*-value** | **Region** | | | | | ***P*-value** |
| --- | --- | --- | --- | --- | --- | --- | --- | --- | --- |
|  | **Urban**  **(n= 8491)** | **Rural**  **(n= 6784)** |  | **East**  **(n=6224)** | **West**  **(n=1993)** | **South**  **(n=2994)** | **North**  **(n=1599)** | **Central**  (**n=2465)** |  |
| Age | | | 0.518 |  | | | | | 0.191 |
| 65- 79 years | 2878 (33.9) | 2336 (34.4) |  | 2128 (34.2) | 634 (31.8) | 1046 (34.9) | 552 (34.5) | 854 (34.6) |  |
| 80- 89 years | 2191 (25.8) | 1776 (26.2) |  | 1583 (25.4) | 520 (26.1) | 781 (26.1) | 414 (25.9) | 669 (27.1) |  |
| ≥ 90 years | 3422 (40.3) | 2672 (39.4) |  | 2513 (40.4) | 839 (42.1) | 1167 (39.0) | 633 (39.6) | 942 (38.2) |  |
| Sex | | | 0.003 |  |  |  |  |  | 0.239 |
| Male | 3837 (45.2) | 2903 (42.8) |  | 2699 (43.4) | 909 (45.6) | 1303 (43.5) | 731 (45.7) | 1098 (44.5) |  |
| Female | 4654 (54.8) | 3881 (57.2) |  | 3525 (56.6) | 1084 (54.4) | 1691 (56.5) | 868 (54.3) | 1367 (55.6) |  |
| Household income (RMB) | | | <0.001 |  |  |  |  |  | <0.001 |
| ＜10000 | 1983 (23.4) | 2748 (40.5) |  | 2102 (33.7) | 503 (25.2) | 983 (32.8) | 221 (13.8) | 922 (37.4) |  |
| 10001–30000 | 1413 (16.6) | 1823 (26.9) |  | 1137 (18.3) | 525 (26.3) | 670 (22.4) | 252 (15.8) | 652 (26.5) |  |
| ＞30000 | 5095 (60.0) | 2213 (32.6) |  | 2985 (48.0) | 965 (48.4) | 1341 (44.8) | 1126 (70.4) | 891 (36.1) |  |
